# The Influence of Early Life Experience on Visceral Pain

**DOI:** 10.3389/fnsys.2018.00002

**Published:** 2018-01-26

**Authors:** Isabella M. Fuentes, Julie A. Christianson

**Affiliations:** Department of Anatomy and Cell Biology, University of Kansas Medical Center, Kansas City, KS, United States

**Keywords:** early life stress, neonatal maternal separation (NMS), visceral hypersensitivity, hypothalamic-pituitary-adrenal (HPA) axis, CRF, chronic pelvic pain

## Abstract

Pain is the most reported and troublesome symptom of nearly all functional disorders affecting the genitourinary and gastrointestinal organs. Patients with irritable bowel syndrome (IBS), interstitial cystitis/painful bladder syndrome (IC/PBS), vulvodynia, and/or chronic prostatitis/chronic pelvic pain syndrome (CP/CPPS; collectively termed chronic pelvic pain syndromes) report pain severe enough to impact quality of life and often suffer from symptoms of or are diagnosed with more than one of these syndromes. This increased comorbidity between chronic pelvic pain syndromes, and with pain disorders of disparate body regions, as well as with mood disorders, can be influenced by disruptions in the hypothalamic-pituitary-adrenal (HPA) axis, which regulates the response to stress and influences the perception of pain. Experiencing trauma, neglect, or abuse in early life can permanently affect the functioning of the HPA axis. As such, a significant proportion of patients suffering from comorbid chronic pelvic pain syndromes report a history of early life stress or trauma. Here we will report on how these early life experiences influence chronic pelvic pain in patients. We will also discuss various rodent models that have been developed to study this phenomenon to understand the mechanisms underlying HPA axis dysfunction, as well as potential underlying mechanisms connecting these syndromes to one another.

## Introduction

The International Association for the Study of Pain (IASP) defines “pain” as an “unpleasant sensory and emotional experience associated with actual or potential tissue damage or described in terms of such damage” (Loeser et al., 1994). A particularly vexing type of pain for both patients and healthcare providers is chronic urogenital or pelvic pain: pain that is localized to the lower abdomen and the pelvic and perineal regions. Chronic pelvic and urogenital pain is common and debilitating, and is the foremost complaint of patients suffering from functional gastrointestinal disorders (e.g., irritable bowel syndrome; IBS) and genitourinary disorders (e.g., interstitial cystitis/painful bladder syndrome; IC/PBS); moreover, this type of pain is idiopathic, meaning the etiologies of these painful disorders are unknown and are not associated with identifiable infectious, anatomical, metabolic, or other organic pathologies. The mechanisms of chronic visceral pain are poorly understood, in part due to its diffuse and poorly localized nature that often involves two or more visceral organs. Additionally, the diverse nature of visceral pain is compounded by multiple factors, including psychosocial stress, sexual dimorphism, and genetic and/or environmental predisposition. These multiple contributing factors make treatment and research efforts, especially the development and study of relevant animal models, challenging.

Despite its multifaceted nature, visceral hypersensitivity has been recognized to occur due to: (1) sensitization of primary sensory afferents innervating the viscera (peripheral sensitization); (2) hyper-excitability of ascending spinal neurons receiving synaptic input from the viscera (central sensitization); and (3) dysregulation of descending pathways that modulate spinal nociceptive transmission (Sengupta, 2009). In this review, we will focus on the influence of early life stress on the descending pathway and feedback loop of the hypothalamic-pituitary-adrenal (HPA) axis, as well as its contribution to chronic pelvic pain of patients suffering from IBS, IC/PBS, vulvodynia, and/or chronic prostatitis/chronic pelvic pain syndrome (CP/CPPS; collectively termed chronic pelvic pain syndromes).

## Hypothalamic-Pituitary-Adrenal Axis

### Central Regulation

**Figure 1:**
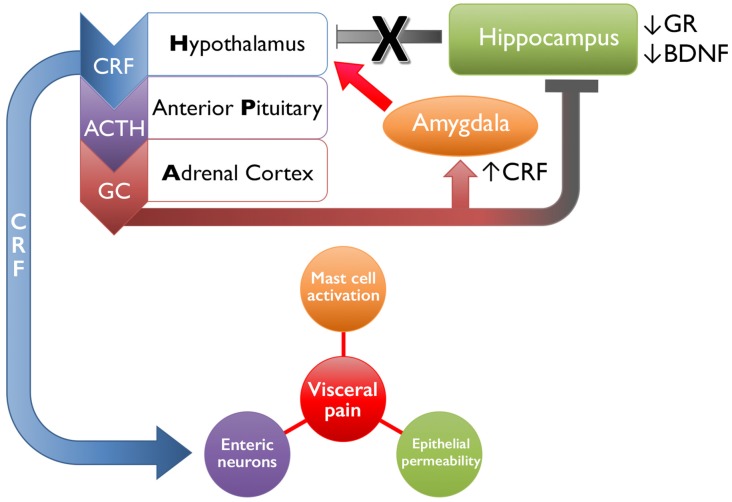
Schematic representation of early life stress-induced changes in limbic regulation of and downstream targets of the hypothalamic-pituitary-adrenal (HPA) axis. Following exposure to an acute stressor, the hypothalamus will release corticotropin-releasing factor (CRF), which signals the anterior pituitary to release adrenocorticotropic hormone (ACTH). The systemic circulation of ACTH initiates the adrenal cortex to release glucocorticoids (GCs, cortisol in humans, corticosterone in rodents). Both GC and CRF will bind to receptors expressed by higher structures within the HPA axis and by limbic structures, including the amygdala and hippocampus, to reduce HPA axis activity and restore homeostasis upon cessation of the stressor. Early life stress disrupts this system by increasing the release of CRF from the hypothalamus and amygdala, as well as decreasing glucocorticoid receptor (GR) and brain-derived neurotrophic factor (BDNF) in the hippocampus, which has a combined effect of increasing positive feedback onto the HPA axis and driving activation. Downstream actions of CRF include increasing mast cell activation and inducing local inflammatory effects, binding onto enteric neurons that can increase colonic motility, and increasing epithelial permeability by disrupting tight junctions. Together these mechanisms drive increased visceral pain in organs affected in irritable bowel syndrome (IBS), interstitial cystitis/painful bladder syndrome (IC/PBS), chronic prostatitis/chronic pelvic pain syndrome (CP/CPPS), and/or vulvodynia.

Stressful events experienced early in life can dramatically alter the functioning of the HPA axis, which regulates the stress response and influences the perception of pain (Heim et al., 1998, 2001; Rao et al., 2008; Tyrka et al., 2008; Mayson and Teichman, 2009; Videlock et al., 2009; schematically shown in Figure 1). Corticotropin-releasing factor (CRF) is the primary initiator of the stress response and, in the brain, is primarily expressed in the paraventricular nucleus (PVN) of the hypothalamus, central nucleus of the amygdala (Bale and Vale, 2004), and Barrington’s nucleus (BN), the pontine micturition center (Imaki et al., 1991; Pavcovich and Valentino, 1995). Under stressful conditions, CRF and arginine vasopressin are secreted from the PVN of the hypothalamus and travel through the hypophysical portal veins to reach the anterior pituitary corticotrophs and induce the release of adrenocorticotropic hormone (ACTH). Systemic circulation of ACTH stimulates the production and release of glucocorticoids (GC, cortisol in humans and corticosterone in rodents) from the adrenal cortex (Herman et al., 2005; Ulrich-Lai and Herman, 2009), which, under normal conditions, initiate an overall immune suppression and decrease CRF and ACTH production through a negative feedback loop (Kageyama and Suda, 2009; Tasker and Herman, 2011).

In addition to CRF, its family members, the urocortins (Ucn1–3), are also produced in stress-related brain regions. Ucn2 and Ucn3 are found in the PVN (Reyes et al., 2001; Venihaki et al., 2004) and Ucn1 is largely expressed in Edinger-Westphal, superior lateral olive, and supraoptic nuclei (Vaughan et al., 1995; Bittencourt et al., 1999). It has been hypothesized that CRF and Ucn1 comprise two separate and functionally intertwined stress-responsive neuronal circuits, as Ucn1 mRNA levels are upregulated in the Edinger-Westphal nucleus following acute pain and/or restraint stress, but on a delayed and longer time scale than CRF is increased in the PVN (Kozicz, 2007). Two G-protein coupled receptors, CRF_1_ and CRF_2_, bind CRF and Ucn ligands with varying affinity. CRF binds CRF_1_ with a 10-fold higher affinity than CRF_2_, Ucn1 binds CRF_1_ and CRF_2_ with equal affinity, and Ucn2 and Ucn3 both preferentially bind CRF_2_ (Bale and Vale, 2004). Opposing roles of CRF_1_ and CRF_2_ in stress-related behaviors have been defined through gene deletion and pharmacological studies. Disruption of CRF_1_ signaling is largely anxiolytic and results in a significant decrease in anxiety-like behaviors (Smith et al., 1998). In contrast, CRF_2_ deletion or blockade increases behavioral indicators of anxiety and prevents homeostatic resolution within HPA axis following a stressful event (Bale et al., 2000, 2002). Stress exposure also promotes CRF release in the central amygdala, a limbic structure involved in memory processing, decision-making, and emotional reactions (Cook, 2004). Chronic GC exposure increases expression of CRF mRNA in the amygdala (Makino et al., 1994, 1999), suggesting that sensitization may be involved in the development of stress-related pathologies (Herman et al., 2012). Studies have also described central activation of CRF receptors mediating stress-related changes in GI function (Van Pett et al., 2000; Reyes et al., 2008). Additionally, studies in children exposed to severe deprivation, neglect, or abuse report lower baseline levels of GCs (Gunnar and Quevedo, 2008; Lupien et al., 2009). It has been hypothesized that this may be due to a downregulation of the HPA axis at the level of the pituitary in response to chronic drive of CRF from the hypothalamus (Fries et al., 2005), or target tissue hypersensitivity to GCs (Yehuda et al., 2006).

Glucocorticoid receptors (GRs) are also abundantly expressed in the hypothalamus and limbic structures and respond to systemic and diurnal release of GCs from the adrenal cortex. The high-affinity mineralocorticoid receptor (MR) can be bound at low circulating levels of GCs, and is thus thought to be important in ambient GC signaling (Dallman et al., 1989) and maintaining the diurnal tone of the HPA axis (Reul and de Kloet, 1985). Tonic feedback within the HPA axis via hippocampal MR is thought to occur by dampening HPA activity during the diurnal trough (van Haarst et al., 1997). The lower-affinity GR is bound during diurnal peaks and spikes in GCs following an acute stressor (Reul and de Kloet, 1985; De Kloet et al., 1998). GR is richly expressed throughout the hippocampus and the prefrontal cortex, limbic structures that are implicated in negative feedback regulation of the HPA axis (Herman, 1993; Herman et al., 2012). Through electrical and chemical stimulation, genetic manipulation, and lesion studies, these brain regions have been shown to be responsible for negative feedback inhibition of the stress response through GR binding, likely in parallel due to innervation of common subcortical targets (Radley and Sawchenko, 2011; Herman et al., 2012). Both MR and GR are highly expressed in hippocampal neurons that, when activated, shut down the HPA axis following the resolution of stressful stimuli by glutamatergic input to GABAergic PVN relays within the hypothalamus (Cho and Little, 1999; Kohara et al., 2001; Numakawa et al., 2009). Negative feedback inhibition of the HPA axis from limbic circuitry is programmed during development and can permanently alter HPA axis function, making the limbic system particularly vulnerable to adversity early in life (Perry and Pollard, 1998; Vázquez, 1998; Bremne and Vermetten, 2001; Heim and Nemeroff, 2002; Teicher et al., 2002, 2003; Rao et al., 2008; Rincón-Cortés and Sullivan, 2014).

### Peripheral Targets

CRF_1_ and CRF_2_ are also widely distributed in the periphery, where ligand-binding activation influences evolutionarily conserved mammalian physiological mechanisms that mediate the return to homeostasis following a stressful event. In the rat colon, CRF_1_ immunoreactivity was observed in the mucosal layer, primarily in cells of an inflammatory nature, and in the myenteric plexus and submucosal plexus; whereas, CRF_2_ immunoreactivity was observed on the luminal surface of goblet cells and in blood vessels located in the submucosa, but not in the enteric innervation (Chatzaki et al., 2004). Expression of CRF_1_ has been confirmed in submucosal and myenteric neurons of the human colon and by macrophages in the lamina propria (Yuan et al., 2012). Exogenous application of CRF or plasma from IBS patients stimulated contractility of explanted rat colon, the latter of which could be blocked by CRF_1_, but not CRF_2_, antagonist pretreatment (Buckley et al., 2014). In the same study, blocking interleukin (IL)-6 or CRF alleviated the increased gastrointestinal motility and improved visceral pain thresholds in a Wistar-Kyoto rat model of IBS. Increased transepithelial resistance and mucosal-to-serosal flux was observed in porcine ileus explants following exposure to increasing amounts of CRF, which was blocked by a non-selective CRF_1/2_ antagonist or a mast cell stabilizer (Overman et al., 2012). Feline urothelial cells also express functionally-active CRF_1_ and CRF_2_, along with their intrinsic ligands CRF and Ucn1 (Hanna-Mitchell et al., 2014). Samples from cats with feline interstitial cystitis, widely accepted as the most relevant preclinical model of IC, demonstrated alterations in CRF receptor expression and functioning, suggesting an etiological role for CRF signaling in this disorder. In human peripheral tissues, CRF_2_ is expressed in high levels in the skin and muscles (skeletal, smooth and cardiac), whereas CRF_1_ is expressed in tissues such as adrenal, adipose tissue, the gonads, endometrium, myometrium, placenta, skin, spleen, and specific cells of the immune system (Hillhouse and Grammatopoulos, 2006).

One downstream target of the HPA axis that has been implicated in nearly all chronic pelvic pain syndromes is the mast cell. Mast cells are multifunctional immune cells that express high-affinity immunoglobulin E receptors and release potent inflammatory mediators including, but not limited to, leukotrienes, cytokines, serotonin, histamine, and proteases, such as tryptase (Ren et al., 1998). Involved in the innate immune response, hematopoietic progenitors of mast cells develop in the bone marrow and are recruited to the peripheral tissues, primarily those interfacing with the environment (e.g., the respiratory-, gastrointestinal-, and genitourinary tracts), where they take up residence and undergo maturation following a complex network of signaling and transcription factors (Tore et al., 2001; Beghdadi et al., 2011). Mast cells are highly responsive to activation of the HPA axis, as they express five isoforms of the CRF_1_ receptor, a single isoform of the CRF_2_ receptor, and contain one of the largest peripheral stores of CRF (Theoharides et al., 2004). The hallmark form of mast cell activation is through degranulation and release of their intracellular stores; however, stress can induce release of cytokines and growth factors in the absence of partial or complete degranulation (Theoharides et al., 2004; Anand et al., 2012). As mast cells are often found in close proximity to nerves and their contents are known to act on nociceptive fibers, it is strongly asserted that mast cells play a key role in peripheral sensitization in chronic pain. Biopsies of affected tissues from patients with IBS (Barbara et al., 2004), IC/PBS (Anand et al., 2012), vulvodynia (Goetsch et al., 2010), and CP/CPPS (Done et al., 2012) have all shown increased mast cell infiltration, degranulation, and/or exuded contents.

## Impact of Early Life Stress or Insult on the HPA Axis

Exposure to early life stress or trauma is a significant risk factor for developing HPA abnormalities and associated chronic pain syndromes (Anand, 1998). The national rate of child maltreatment in the United States has consistently risen over the past decade (U.S. Department of Health and Human Services, Administration for Children and Families, and Administration on Children, Youth and Families, Children’s Bureau, 2012) and exposure to adverse childhood experiences (ACEs) significantly increases the risk of developing chronic disease and disability later in life (Gilbert et al., 2015). Although medical advancements have allowed for prematurely-born babies to survive at increasingly earlier gestational stages, prolonged stays in the neonatal intensive care unit (NICU) provide chronic exposure to numerous stressors, including repeated invasive procedures and prolonged periods of maternal separation (Simons et al., 2003). A preterm neonate in the NICU is subjected to approximately 16 interventional procedures per day, 10 of which are considered to be painful (Carbajal et al., 2008). The likelihood of NICU admission is higher for full-term babies born to women aged 40–44, an age group whose birth rate rose 4% in 2015 (Martin et al., 2017). If current trends continue, increasing rates of childhood adversity and stress will add extra layers of complexity onto already prevalent and hard-to-treat chronic pain disorders.

Rodent models incorporating early life stress or peripheral insult have demonstrated disrupted functioning of the HPA axis. Neonatal maternal separation (NMS) in rodents has been used for several decades as a model of early life stress that significantly impacts the functioning of the HPA axis (McIntosh et al., 1999; Romeo et al., 2003; Daniels et al., 2004; Millstein and Holmes, 2007). In rats, the neonatal period between postnatal day 1 (P1) through P14 is critical for neurological development. In humans, this period begins prenatally and lasts until age 5 (Perry and Pollard, 1998); the nervous system is exceptionally pliant to nurturing and adverse events during this critical window of development (Teicher et al., 2002). Maternal deprivation in rats lowered GR expression in the hippocampus and cortex, resulting in protracted responses to acute stress and deficient GC feedback in rats (Ladd et al., 2004). Altered hypothalamic and limbic CRF receptor and GR expression has also been reported (Ladd et al., 2004; Plotsky et al., 2005; Aisa et al., 2008; O’Malley et al., 2011a). These animals also had increased corticosterone levels (O’Mahony et al., 2009) and prolonged ACTH release following stressful events (Romeo et al., 2003; Ladd et al., 2004; Plotsky et al., 2005; Aisa et al., 2008); induced depressive behaviors including heightened anxiety behaviors in an open field area (McIntosh et al., 1999; Daniels et al., 2004; Millstein and Holmes, 2007; O’Malley et al., 2010); and increased visceromotor response (VMR) to colorectal balloon distention (CRD; Coutinho et al., 2002; Zhang et al., 2009; O’Malley et al., 2010; Moloney et al., 2012). The hyperalgesia was exacerbated following exposure to water avoidance stress (WAS) and was prevented by a preemptive administration of a selective CRF_1_ antagonist (Schwetz et al., 2005), indicating that CRF may play a critical role in the development of visceral hypersensitivity. Altered functioning of the HPA axis likely drove the heightened sensitivity to acute stressors and anxiety-provoking situations in NMS rats (Taché et al., 2001; O’Malley et al., 2011a), as CRF mRNA and CRF_1_ immunoreactivity were both elevated following NMS in PVN, amygdala, and locus coeruleus (Plotsky et al., 2005). Moreover, NMS rats displayed significantly higher levels of c-Fos expression in the cingulate cortex and superficial and deeper laminae of the spinal cord in response to colorectal distention than did naïve rats (Chung et al., 2007; Ren et al., 2007), suggesting that early life stress likely induced functional changes in the central descending modulatory system that may have contributed to hyperalgesia (Sengupta, 2009).

Rodent models of peripheral inflammation or insult during neonatal development also demonstrate altered HPA axis functioning in adulthood. Rat pups that were subjected to gastric suctioning from postnatal day 2–12 developed colorectal sensitivity as adults that could be mitigated by CRF_1_ antagonist treatment prior to suctioning (Smith et al., 2007). Our lab demonstrated that neonatal vaginal irritation (NVI) in mice resulted in vaginal and colorectal hypersensitivity that could also be prevented by CRF_1_ antagonist treatment at the time of insult (Pierce et al., 2015). Many additional studies have used neonatal peripheral inflammation or insult in rodents as preclinical models of chronic pelvic pain disorders with or without effects on multiple organ systems. These include neonatal bladder irritation with zymosan (Randich et al., 2006; Miranda et al., 2011) or neonatal colon irritation with mustard oil (Al-Chaer et al., 2000; Christianson et al., 2010), acetic acid (Winston et al., 2007), or colorectal distension (Al-Chaer et al., 2000; Lin and Al-Chaer, 2003; Wang et al., 2008). All of these studies reported increased visceral sensitivity with alterations in central and/or peripheral nociceptive processing; however, none directly interrogated the potential role of altered HPA axis regulation or output.

## Clinical Evidence of Early Life Stress-Related Visceral Pain Syndromes

Experience of early life adverse childhood events has been linked to maladjusted stress response in adulthood, and can serve as a risk factor for developing mood and functional pain disorders later in life (Grunau et al., 1994; Anand, 1998; Whitfield and Grunau, 2000; Mayson and Teichman, 2009; O’Malley et al., 2011a,b; Maniam et al., 2014). Accordingly, patients suffering from chronic pelvic pain syndromes commonly report having endured early life stress and often experience stress-related symptom onset or increased severity. Symptoms of more than one syndrome, as well as comorbid mood disorders, are often experienced in chronic pelvic pain patients (Green et al., 2010; Warren et al., 2011b; Potts and Payne, 2012; Bullones Rodríguez et al., 2013; Suskind et al., 2013), complicating already less-than-ideal treatment strategies and compounding the negative impact on quality of life. In this section, we will discuss evidence of early life-induced symptomology and the potential role of altered HPA axis signaling in four common chronic pelvic pain syndromes.

### Irritable Bowel Syndrome

IBS is the most commonly diagnosed chronic pelvic pain disorder, as well as functional gastrointestinal disorder. IBS is characterized by chronic or recurrent abdominal pain or discomfort and altered bowel habits. Symptom onset or exacerbation is often triggered by stress in patients suffering from IBS (Mayer et al., 2001; Blanchard et al., 2008; Dufton et al., 2008; Stasi et al., 2012; Fond et al., 2014). A reported history of early life stress, such as premature birth, neglect, abuse, loss of a parent, or parental discord, is a significant risk factor for developing IBS in adulthood (Barreau et al., 2007; Chitkara et al., 2008; Videlock et al., 2009). Premature birth or low birth weight increases the risk of developing IBS, in part due to a developmentally immature gastrointestinal system at birth (Berseth, 1989; Bengtson et al., 2006). Gastric suctioning shortly after birth can also increase the risk of developing functional gastrointestinal disorders (Anand et al., 2004).

Improper functioning of the HPA axis, particularly over-activity, has been noted in sub-populations of IBS sufferers. In a study that investigated the impact of childhood trauma on HPA axis responsiveness in IBS patients, subjects that reported early adverse life events had significantly higher salivary cortisol levels following sigmoidoscopy than control patients (Videlock et al., 2009). In those patients with IBS, a faster resolution of stress-induced cortisol correlated with lower symptom severity and higher quality of life ratings. Furthermore, over-activation of the HPA axis was more related to a history of early life adverse events than to the presence of IBS, suggesting that exposure to early life stress alone may not be sufficient for the manifestation of IBS. In a separate study that did not interrogate the role of early life stress exposure, female IBS patients had significantly lower basal ACTH levels, but higher basal and stimulated plasma cortisol levels. They also displayed a positive correlation between plasma cortisol and reported anxiety levels prior to sigmoidoscopy (Chang et al., 2009). Based on preclinical rodent studies, increased CRF/CRF_1_ signaling in both the brain and colon has been proposed to contribute towards comorbid anxiety/depression in female diarrhea-predominant IBS patients (Taché et al., 2005). Clinical studies looking at the effectiveness of CRF_1_ antagonist treatment have had mixed success in patients. An initial study reported no effect of CRF_1_ antagonist on influencing colonic transit time, stool frequency, or consistency in female diarrhea-predominant IBS patients (Sweetser et al., 2009). Reported anxiety and depression scores were not different between placebo and treatment groups; however, the authors state that 7/30 patients reported anxiety scores that indicate significant anxiety. Considering the role of comorbidity in HPA axis over-activity, it would have been informative to determine whether the CRF_1_ antagonist was more or less effective in these highly anxious patients. A later study addressed this relationship by measuring the effectiveness of a CRF_1_ antagonist on mediating the functional connectivity of the emotional-arousal circuit using fMRI prior to the expectation of abdominal pain (Hubbard et al., 2011). In this study, the CRF_1_ antagonist, relative to placebo, significantly reduced the blood oxygen level-dependent signal in the hypothalamus in IBS patients with average or high levels of anxiety during expectation of abdominal pain. The authors suggested that their findings, when compared to previous, negative outcomes with CRF_1_ antagonists, support the idea that targeting the CRF/CRF_1_ signaling pathway may only be beneficial in a subset of patients who also display stress sensitivity, anxiety, and hyper-responsiveness of the HPA axis. These studies emphasize the etiologic heterogeneity underlying IBS, despite similar symptomology. Further understanding of IBS and comorbidities will be essential in designing appropriate, personalized treatment strategies.

Due to the significant impact of early life stress on IBS development, NMS is a frequently used preclinical model for understanding the mechanisms underlying early life stress-induced gastrointestinal hypersensitivity and dysfunction. Adult rodents that were exposed to NMS exhibit many of the same colorectal sensitivities and functional and neuroimmune abnormalities observed in human cases. These include increased growth factor and cytokine expression, such as NGF, IL-6, IL-1β, IL-2, IL-4, IL-10, and interferon (IFN)-γ, as well as infiltration of mast cells, in the distal colon, all of which can sensitize peripheral nociceptors and enhance visceral perception (Barreau et al., 2004a,b; Daniels et al., 2009; van den Wijngaard et al., 2009, 2012; O’Malley et al., 2011a,b; Lennon et al., 2013). While these studies have all been performed in rat models, studies from our lab using mice have revealed little to no effect of NMS on colorectal sensitivity, cytokine expression, or mast cell infiltration, with the exception of increasing susceptibility to trinitrobenzene sulfonic acid (TNBS)-induced colitis in male mice (Fuentes et al., 2016; Pierce et al., 2016). One other study on NMS in mice reported an increase in colonic hypersensitivity; however, this model also incorporated unpredictable maternal stress with unpredictable NMS, which likely altered the outcomes of this study, as it relates to our findings (Moloney et al., 2012). Other recent studies have highlighted the influence that the gut microbiota has on stress-related gastrointestinal disorders (Moloney et al., 2016), which can be impacted by age, sex, diet, genotype, and environment (Laukens et al., 2016), making it difficult to compare the outcomes of similar studies even in the same strain of mice. It is likely that contributing variables outside of species, sex, strain, and stressor-type will emerge as this field continues to move forward.

### Interstitial Cystitis/Painful Bladder Syndrome

The chief complaints of IC/PBS patients include idiopathic pelviperineal pain and increased urinary urgency and frequency. Diagnosis of IC/PBS is largely symptom-based, particularly irritative voiding and referred lower urinary tract pain, after excluding other pathologies that mimic symptoms of IC/PBS (e.g., urinary tract infection; Hanno et al., 2011). As with other functional pain disorders, IC/PBS patients are more likely to have a history of early life stress or adversity. In a study of 87 female patients with IC/PBS, just over half reported a history of abuse, the majority of which occurred during childhood or adolescence (Peters et al., 2008). In another study of 207 female IC/PBS patients, 24% reported sexual abuse prior to the age of 17, compared to 15% in the control group. Those IC/PBS patients with a history of abuse had greater comorbid depression, anxiety, and mental functioning, as well as greater pain, but not functional symptoms, than those who did not report abuse (Nickel et al., 2011). Patients with IC/PBS who reported childhood trauma perpetuated by someone with whom they had a close relationship had a greater degree of comorbid anxiety, despite similar urogenital symptom severity, compared to IC/PBS patients without a history of abuse (Chiu et al., 2017). Multiple studies have reported that patients with IC/PBS have a higher incidence of childhood bladder problems, including recurrent urinary tract infections (Jones and Nyberg, 1997; Peters et al., 2008).

Similar to the aforementioned fMRI study illustrating increased functional connectivity in the emotional-arousal circuit of IBS patients, multiple studies have investigated changes in brain connectivity in patients with IC/PBS. Patients with more widespread pain, affecting multiple and disparate parts of the body outside of the pelvis, had greater increases in gray matter volume and functional connectivity of sensorimotor and insular cortices (Kutch et al., 2017). These changes also correlated with decreased physical and mental function. A separate study also correlated widespread pain with increased comorbid anxiety and depression and a lesser quality of life (Lai et al., 2017). While these studies do not directly address the role of the HPA axis or early life stress, the evidence linking these occurrences with comorbidity of mood disorder suggest that these patients may have a higher likelihood of early adverse events and/or increased activation of the HPA axis. In support of the latter, molecular and histological evidence of increased CRF signaling is present in tissue samples, serum, and/or urine from IC/PBS patients. Tissue biopsies from IC/PBS patients have revealed increased mast cell infiltration (Kastrup et al., 1983; Christmas and Rode, 1991; Spanos et al., 1997; Peeker et al., 2000; Tomaszewski et al., 2001; Larsen et al., 2008) and close proximity to densely-populated substance P (SP)-immunopositive nerve fibers (Pang et al., 1995). Mast cell tryptase was elevated in IC/PBS urine (Boucher et al., 1995; Okragly et al., 1999) and elevated NGF levels in seminal plasma have been demonstrated to be directly correlated with pain severity (Miller et al., 2002; Watanabe et al., 2011). Elevated concentrations of NGF, histamine, and pro-inflammatory cytokines have also been observed in IC/PBS patients’ serum (Jiang et al., 2013) and urine (Yun et al., 1992; Lotz et al., 1994; Jacobs et al., 2010; Corcoran et al., 2013).

CRF is not only involved in inflammation and pain signaling in the context of IC/PBS, BN, located in the dorsolateral pontine tegmentum, is central to the micturition pathway and uses CRF as a neurotransmitter within this relay (Vincent and Satoh, 1984; Sakanaka et al., 1987; Valentino et al., 1995). Neurons from the BN project to the lumbosacral parasympathetic nucleus, where preganglionic neurons innervate the pelvic viscera, and send collateral branches to the locus coeruleus (Sasaki, 2005; Valentino et al., 2011). Information relayed by bladder afferents from both the spinal cord, as well as the periaqueductal gray region (Blok et al., 1995; Ding et al., 1997; Rouzade-Dominguez et al., 2003b), stimulates BN neurons to induce increases in bladder pressure, cause bladder contraction, inhibit the external urethral sphincter, and relax the urethra (Sasaki, 2005). Activation of the locus coeruleus increases arousal and shifts the mode of attention to modify behavior to best coordinate visceral functions, such as voiding, through dense projections throughout the cortex (Berridge and Waterhouse, 2003; Aston-Jones and Cohen, 2005). Neurons in the BN have been shown to be sympathetically linked to and able to influence the distal colon, genitals, and bladder, allowing for co-regulation of multiple organ systems under certain conditions, such as stress (Marson, 1995; Marson and McKenna, 1996; Rouzade-Dominguez et al., 2003a). This organization may contribute towards the comorbidity of multiple pelvic visceral symptoms as CRF may play a role in regulating bladder or colonic motility as a component of the stress response (Valentino et al., 1999). Repeated social defeat stress in adult rats has been shown to increase CRF expression within BN (Kiddoo et al., 2006; Wood et al., 2009) and promote urine retention (Wood et al., 2009). Excitatory effects of CRF on colonic motoneurons may contribute to stress-related increases in colonic motility and symptoms of IBS (Pavcovich et al., 1998; Valentino et al., 1999); however, *in vivo* cystometry studies have suggested both excitatory and inhibitory effects of CRF on micturition by evaluating the effects of CRF and CRF antagonists administered systemically or intrathecally (Klausner and Steers, 2004; Klausner et al., 2005; Kiddoo et al., 2006). Treatment with CRF_1_ antagonist reduced both bladder filling and micturition volumes that were initially increased due to intrathecal administration of CRF or Ucn2 (Kiddoo et al., 2006). Klausner et al. (2005), however, observed the opposite effect. CRF administration decreased micturition volume in normal Wistar rats, and intrathecal administration of astressin, a non-selective CRF_1_/CRF_2_ antagonist, increased void volumes of high-anxiety Wistar-Kyoto rats (Klausner et al., 2005). The exact role of CRF expression in BN in humans, particularly as it relates to IC/PBS, has yet to be elucidated, however these studies and others suggest it may play a pivotal role in stress-related symptomology.

Preclinical models incorporating early life stress and bladder inflammation have been used to investigate possible mechanisms underlying IC/PBS. Rats treated with intravesicular zymosan, an irritant component of the yeast cell wall, on postnatal days 14 and 16 displayed increased micturition frequency and heightened VMR and arterial blood pressure during urinary bladder distension (UBD) following reinflammation with zymosan as adults (Randich et al., 2006). Later investigations into this model revealed disruption of descending inhibitory pathways (DeBerry et al., 2007) and increased plasma extravasation and neuropeptide release after intravesicular mustard oil application (DeBerry et al., 2010). Studies from our laboratory showed that NMS in female mice increases the VMR during UBD both at baseline and following an acute exposure to WAS (Pierce et al., 2016). Bladder mast cell degranulation and pro-inflammatory gene expression profiles were significantly increased in female NMS mice, along with molecular evidence of altered hippocampal input onto the HPA axis. Stress-induced bladder hypersensitivity, vascular permeability, and upregulated peripheral expression of TNF-α and IL-10 in adult rat models have been described as being driven by a CRF_2_ rather than a CRF_1_-mediated mechanism (Robbins and Ness, 2008; Huang et al., 2009; Boucher et al., 2010; Novembri et al., 2011). Intrathecal administration of a CRF_2_ antagonist prior to UBD attenuated unpredictable footshock-induced urinary bladder hypersensitivity as measured by VMR (Robbins and Ness, 2008).

### Chronic Prostatitis/Chronic Pelvic Pain Syndrome

CP/CPPS is characterized by chronic, idiopathic pain in the lower abdomen, rectum, perineum, prostate, penis, and/or testicles, with or without urinary symptoms (Duloy et al., 2007; Murphy et al., 2009), and is diagnosed symptomatically due to the lack of associated pathology (Pontari and Ruggieri, 2004). CP/CPPS has detrimental effects on quality of life, comparable to myocardial infarction, angina, Crohn’s disease, and diabetes (Murphy et al., 2009; Strauss and Dimitrakov, 2010). Little is known regarding the influence of early life stress or adversity on the later development of CP/CPPS; however, the significant overlap with IC/PBS and susceptibility to stress suggests a potential early life component to the disorder. Additionally, because male patients have been reported to experience significant psychological disturbances (McNaughton Collins et al., 2001; Tripp et al., 2006; Anderson et al., 2008, 2009; Nickel et al., 2008; Shoskes et al., 2009; Ahn et al., 2012; Riegel et al., 2014) similar to female patients that have experienced early life stress (Heim et al., 1998, 2002; Mayson and Teichman, 2009; Nickel et al., 2010; Warren et al., 2011a, b), it has been hypothesized that men with CP/CPPS may have similar histories. Findings from the Multi-Disciplinary Approach to the Study of Chronic Pelvic Pain (MAPP) Research Network reported that both male and female patients suffering from urologic chronic pelvic pain syndromes (UCPPS; specifically, CP/CPPS in males and IC/PBS in females) exhibit greater psychological distress, poorer coping, higher levels of current and lifetime stress, and more widespread pain symptoms that contribute to poorer self-reported quality of life than sex- and education-matched healthy controls. The study goes on to report that individuals living with UCPPS experience greater incidences of early life and adult trauma compared to healthy controls (though women more significantly than men; Naliboff et al., 2015). To our knowledge, only one study has looked explicitly at the prevalence of early childhood trauma and developing CP/CPPS in adulthood: the Boston Area Community Health (BACH) survey examined the relationship between sexual, physical, or emotional abuse and symptoms suggestive of CP/CPPS. Hu et al. (2007) concluded that among the 2301 men recruited, reporting abuse increased the odds of also presenting symptoms suggestive of CP/CPPS, though greater frequency of both childhood and adult abuse were associated with increased prevalence of CP/CPPS symptomology. Furthermore, if a subject reported more than one type of abuse, he was at increased odds of reporting both increased pain and urinary scores of the NIH chronic prostatitis symptom index (CPSI), suggesting a cumulative effect (Hu et al., 2007).

As with the other chronic pelvic pain syndromes, the etiology underlying CP/CPPS is largely unknown, although stress has been shown to worsen or bring on symptoms. Evidence of altered HPA axis functioning has been demonstrated primarily by increased mast cell activation in the affected tissue. Though studies have reported varied degrees of mast cell degranulation and activation, CP/CPPS biopsies were observed having altered granular structure (Theoharides et al., 1990) and decreased numbers of intact mast cells (Amir et al., 1998), suggesting that mast cell activation without complete degranulation and increased rate of complete degranulation could occur in CP/CPPS. Concentrations of tryptase and carboxypeptidase A (CPA3), a marker of mast cell activation, were increased in urine samples from CP/CPPS patients (Roman et al., 2014). Mast cell tryptase and NGF levels were also increased in expressed prostatic secretions from patients (Done et al., 2012). Furthermore, Anderson et al. (2008, 2009) have reported that men with CP/CPPS, compared to healthy controls, have a greater slope of waking cortisol response (Anderson et al., 2008) and delayed ACTH release correlating with significant psychological disturbances (phobic anxiety, perceived stress, depression, etc.) in response to an acute stress (Anderson et al., 2009), suggesting altered HPA axis function.

The majority of published rodent models of CP/CPPS are centered around experimental infection; injection of exogenous antigens, androgens, or irritants (Pierce and Christianson, 2015); or invasive surgery (Vykhovanets et al., 2007) and, as such, are largely undefined in their mechanism of CP/CPPS development. We have recently begun investigating the use of NMS in mice as a model of CP/CPPS. Adult male NMS mice display significant perigenital mechanical hypersensitivity, as well as significantly increased mast degranulation in the prostate and bladder (Fuentes et al., 2015; current issue). This model also displays increased micturition frequency and output and molecular evidence of altered HPA functioning (current issue). Further investigation of NMS in mice will hopefully provide a more clinically relevant rodent model of CP/CPPS for investigation of potential therapies and interventions.

### Vulvodynia

Vulvodynia presents clinically as vulvar discomfort, most often as a burning pain, with no identifiable pathological or neurological evidence of disease (Moyal-Barracco and Lynch, 2004). Although there is variability in clinical presentation, the hallmark symptom is allodynia and/or burning, stinging, or itching of the vulva, vestibule, or vaginal canal (McKay, 1988; Edwards, 2004). Early life adverse events are strongly linked with an increased likelihood of vulvodynia in adulthood and are attributed to dysfunctional regulation of the HPA axis. Vulvodynia was strongly associated with a sense of danger or abuse, particularly by a primary family member, during childhood (Harlow and Stewart, 2005). A separate study did not find an increase in childhood abuse; however, parental divorce was more common among women with vulvodynia when compared to controls (Plante and Kamm, 2008). In a survey of women diagnosed with IC/PBS, 63% reported vulvar pain and 29% reported a history of abuse (Carrico et al., 2009). Women with vulvodynia have blunted serum cortisol cycles (Ehrström et al., 2009) and, like with other chronic pelvic pain syndromes, symptom severity is increased following acute stress exposure (Gordon et al., 2003). Defective regulation of the downstream inflammatory response is also observed as increased mast cell degranulation and infiltration within vestibular biopsies when compared to controls (Bornstein et al., 2008). Mast cell-derived heparanase is increased in the vestibule of vulvodynia patients (Bornstein et al., 2008), where it serves to degrade the heparin sulfate component of basement membranes and the extracellular matrix as needed for leukocyte infiltration (Goldberg et al., 2013). Age-related changes have been observed, as mast cell activation increases in post- compared to pre-menopausal vulvodynia patients, despite similar expression levels of estrogen receptor α and evidence of neural hyperplasia (Leclair et al., 2013). Increases in proinflammatory cytokines IL-1 and TNF-α have also been reported (Foster and Hasday, 1997; Jeremias et al., 2000), both of which have been demonstrated to be produced by activated mast cells (Galli et al., 2005). Moreover, decreased IL-1 receptor antagonist (IL-1RA) activity has been observed in vulvodynia patients, and has been associated with increased IL-1 beta activity, which has been observed in tissues sampled from patients (Foster et al., 2007). Inflammatory challenge in patients resulted in blunted IL-1RA response (Gerber et al., 2002). The prevention of stress-induced elevations in IL-1β and associated increase of HPA axis activity by pretreatment with IL-1RA (Gądek-Michalska et al., 2011), suggests that peripheral cytokines can influence central perceptions of pain through limbic control.

Despite the clinical prevalence of vulvodynia, few animal models have been published investigating preclinical mechanisms possibly underlying this disorder. Animal models have exploited both the hormonal and allodynic characteristics of vulvodynia by observing peripheral neuron sprouting under low estrogenic conditions (Bhattacherjee et al., 2013), vulvar allodynia following repeated vulvovaginal fungal infection (Farmer et al., 2011), and oxazolone-induced delayed-type contact hypersensitivity of the vulva (Martinov et al., 2013). We have developed two separate preclinical models of vulvodynia using NMS (Pierce et al., 2014) and NVI with zymosan (Pierce et al., 2015). Both models develop significant vaginal hypersensitivity in adulthood with associated increases in HPA axis gene expression levels and/or output. As mentioned above, the vaginal hypersensitivity in the NVI model was prevented by pretreating with CRF_1_ antagonist prior to zymosan treatment, verifying the involvement of the HPA axis in the development of vaginal hypersensitivity (Pierce et al., 2015).

## Centralized Pain Phenotype

Obvious similarities are present between these previously discussed syndromes, including a lack of overt pathology (with the exception of IC) and disruption of proper functioning of the HPA axis. Patients diagnosed with one of these disorders are very often diagnosed with, or present with symptoms of, additional functional pain syndromes and/or mood disorders, such as anxiety, panic disorder, or depression (Bullones Rodríguez et al., 2013). This high rate of comorbidity, as well as similar symptomology, has led to the hypothesis that these syndromes are different manifestations of a centralized pain phenotype possibly resulting from early life trauma (Nicol et al., 2016). Recent work from the MAPP Research Network has shown that patients with IC/PBS are less likely to have pain restricted to the pelvis (25% of patients) than intermediate pain around the pelvis (37% of patients) or widespread pain throughout the body (38% of patients; Lai et al., 2017). Patients with a greater number of painful body sites also reported higher pain, anxiety, and depression scores, along with worse quality of life. Changes in functional connectivity between relevant brain structures, as well as increased gray matter, have also been correlated with worsened pain and mental function scores (Kutch et al., 2017). These observations support the need for fully phenotyping patients prior to prescribing pharmacological or other interventional treatments. While patients reporting pain only in the affected organ may respond to peripherally-restricted therapies, most patients have multiple comorbidities and may respond more effectively to therapies targeted to the central nervous system. Future work in our lab and others are exploring the role of the central nervous system, and interventions such as exercise, in preclinical models of chronic pelvic pain syndromes to evaluate their efficacy in preventing or alleviating symptomology similar to that seen in humans.

## Conclusion

Patients with chronic pelvic pain disorders commonly report having experienced early life stress, trauma, or peripheral insult, including repeated infections. The strong influence that stress has on initiating and/or exacerbating symptoms associated with pelvic pain disorders further strengthens this relationship between stress and pain, likely due to the influence of the HPA axis. Development of clinical therapies and preclinical animal models should heed this important and influential relationship to improve treatment options and better understand the underlying etiology of these related disorders.

## Author Contributions

IMF and JAC: equal contribution for the literature search and article preparation.

## Conflict of Interest Statement

The authors declare that the research was conducted in the absence of any commercial or financial relationships that could be construed as a potential conflict of interest.
